# Leading Indicators and the Evaluation of the Performance of Alerts for Influenza Epidemics

**DOI:** 10.1371/journal.pone.0141776

**Published:** 2015-10-29

**Authors:** Dena L. Schanzer, Myriam Saboui, Liza Lee, Francesca Reyes Domingo, Teresa Mersereau

**Affiliations:** 1 Centre for Communicable Diseases and Infection Control, Infectious Disease Prevention and Control Branch, Public Health Agency of Canada, Ottawa, Ontario, Canada; 2 Centre for Immunization and Respiratory Infectious Diseases, Infectious Disease Prevention and Control Branch, Public Health Agency of Canada, Ottawa, Ontario, Canada; New York City Department of Health and Mental Hygiene, UNITED STATES

## Abstract

**Background:**

Most evaluations of epidemic thresholds for influenza have been limited to internal criteria of the indicator variable. We aimed to initiate discussion on appropriate methods for evaluation and the value of cross-validation in assessing the performance of a candidate indicator for influenza activity.

**Methods:**

Hospital records of in-patients with a diagnosis of confirmed influenza were extracted from the Canadian Discharge Abstract Database from 2003 to 2011 and aggregated to weekly and regional levels, yielding 7 seasons and 4 regions for evaluation (excluding the 2009 pandemic period). An alert created from the weekly time-series of influenza positive laboratory tests (*FluWatch*, Public Health Agency of Canada) was evaluated against influenza-confirmed hospitalizations on 5 criteria: lead/lag timing; proportion of influenza hospitalizations covered by the alert period; average length of the influenza alert period; continuity of the alert period and length of the pre-peak alert period.

**Results:**

Influenza hospitalizations led laboratory positive tests an average of only 1.6 (95% CI: -1.5, 4.7) days. However, the difference in timing exceeded 1 week and was statistically significant at the significance level of 0.01 in 5 out of 28 regional seasons. An alert based primarily on 5% positivity and 15 positive tests produced an average alert period of 16.6 weeks. After allowing for a reporting delay of 2 weeks, the alert period included 80% of all influenza-confirmed hospitalizations. For 20 out of the 28 (71%) seasons, the first alert would have been signalled at least 3 weeks (in real time) prior to the week with maximum number of influenza hospitalizations.

**Conclusions:**

Virological data collected from laboratories was a good indicator of influenza activity with the resulting alert covering most influenza hospitalizations and providing a reasonable pre-peak warning at the regional level. Though differences in timing were statistically significant, neither time-series consistently led the other.

## Introduction

Many countries have used thresholds of weekly time series of consultation rates for influenza-like-illnesses (ILI) to signal the start and end of the period of seasonal influenza activity. The resulting epidemic period identifies a period where ILI activity is considered in excess of what is normally expected. However these thresholds are often set based only on visual inspection [1]. Recently, researchers have evaluated other sources of real time data such as telehealth [2,3], prescription sales [4] or emergency department visits for specific syndromes commonly associated with ILI [5,6] for the purpose of signalling an emerging influenza epidemic earlier. Others have evaluated time series from social media. Google Flu Trends (GFT) is an application that calibrates the number of web searches for terms associated with ILI to the weekly ILI time series provided by public health surveillance. Because search queries can be processed quickly, the resulting ILI estimates were found to be consistently 1–2 weeks ahead of CDC ILI surveillance reports [7,8]. Time series based on Wikipedia page views have produced similar results [9].

Despite the strong correlations, various issues have been identified. Since the impact of influenza is highly variable, it is not surprising that re-calibration is necessary for Google Flu Trends to track ILI consultation rates, or that significant differences between the two time series were identified [10]. Performance evaluation has often been limited to using proposed standards, to comparing the proposed alert with the ILI based epidemic period, or to an internal validation [11–13]. Since the epidemic grows exponentially for many weeks before the first cases are detected through laboratory testing, or excess morbidity or mortality outcomes are identifiable [11,14], an alert that signals the emergence of an influenza epidemic before the excess is observed has considerable utility that may not be apparent using the ILI based epidemic period as the gold standard.

With these limitations in mind, we aimed to illustrate the insight provided by the cross-validation of an alert against public health oriented criteria rather than simply showing correlation or performing an internal validation. We chose virological data (number and percent of laboratory tests positive for influenza) as the candidate indicator variable, as this data appeared to be the most promising indicator from our national surveillance program, *FluWatch* [15]. As the weekly number of influenza-confirmed hospitalizations has been shown to be a good proxy at the seasonal level for excess morbidity and mortality attributable to influenza [16], we used weekly influenza-confirmed hospitalizations as the validation dataset. We drew upon a number of statistical measures to determine whether the two samples arose from the same distribution, that is, we tested for differences in timing (did laboratory reports or hospital admissions lead or lag?), and shape of the distribution function (which time series has longer tails, or more extreme values?). This approach provides a richer description than the correlation and cross-correlation analyses used elsewhere. Next we aimed to assess the alert period by characteristics of potential interest to hospital resource management and developed 5 criteria: lead/lag and other timing differences; proportion of influenza hospitalizations covered by the alert period; average length of the influenza alert period; continuity of the alert period; and length of the pre-peak alert period.

## Methods

### Sources of data

Hospital discharge records for patients admitted to an acute care hospital with a diagnostic code of influenza, virus identified (J10) were extracted from the Canadian Institute of Health Information (CIHI) patient-specific Discharge Abstract Database (DAD) [17] from April 2004 to March 2011, a period when all DAD participating provinces used the *International Classification of Disease*, *Tenth Modification* (ICD-10) [18] for diagnostic coding. The province of Quebec does not participate in the DAD, hence the DAD includes approximately 75% of all acute care hospital separations in Canada. Hospitalizations were aggregated to weekly and regional levels, yielding 7 seasons (2003/04, 2004/05, 2005/06, 2006/07, 2007/08, 2008/09, 2010/11) and 4 regions (Atlantic Provinces, Ontario, Prairie Provinces, and British Columbia) for evaluation.

The weekly number of respiratory virus identifications for influenza A and B and other viruses as well as the number of tests were obtained from the Public Health Agency of Canada’s *FluWatch* program [19]. Routine surveillance data is collected for each epidemiological week and published in a surveillance report within 2 weeks. Seasons were defined regionally from September to August of the following year. The weekly distribution function was calculated by dividing the weekly number of events by the seasonal total for each region. The weekly positivity rate was calculated by dividing the weekly number of influenza positive tests by the weekly number of tests. The 2009 pandemic period was excluded from the analysis, as laboratory testing initially increased sharply once circulation of a novel strain with pandemic potential was announced in late April of 2009, and subsequently declined substantially over the pandemic period.

### Statistical Analysis

#### Lead-lag and other timing differences

To test for differences in the timing of influenza infections between the two weekly time-series, we performed a two sided t-test using Satterthwaite variance calculation to account for unequal variances for each of the 4 regions and 7 seasons, and reported the mean difference along with Satterthwaite confidence intervals. We used the Folded F test to test for equality of variance, and the two-sample Kolmogorov-Smirnov Test to test for significant differences in the cumulative distribution function (CDF). The two-sample Kolmogorov–Smirnov test is a nonparametric method for comparing the distribution of two samples which is sensitive to differences in location (median), spread (parametric equivalent is the standard deviation) and other shape characteristics such as differences in skewness or heavier tails. Confidence intervals for the Pearson correlation coefficient were calculated using Fisher’s z transformation. Differences in timing and other differences between the CDFs were summarized for the 28 seasons. SAS Enterprise Guide 5.1 [20] was used for the analysis and provides descriptions of these statistics.

#### The alert and evaluation

**Fig 1:**
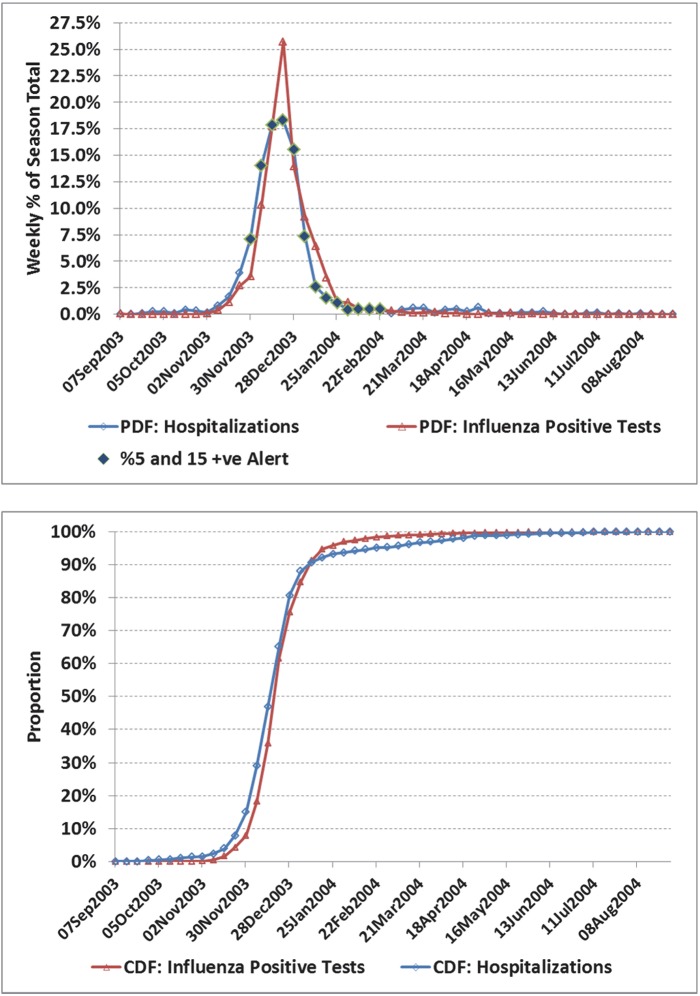
a) Weekly number of influenza positive tests and influenza admissions to hospital for Ontario, 2003/04 season. A novel virus, A⁄Fujian⁄411⁄02 accounted for 95% of the isolates characterized in the 2003/04 season. Alerts based on the 5% positivity rule with 15 positive tests with an adjustment for the 2 week operational delay are indicated against the influenza hospitalization time series as a solid diamond. For example, the alert was first set based on virological data for the week of November 16, with the alert period starting 2 weeks later (week of Nov 30), or 3 weeks before the peak in influenza hospitalizations (week of Dec 21). b) Corresponding cumulative distribution functions (CDF). Hospital admissions led by an average of only 1 day. Despite the close alignment during the period peak influenza activity, a comparison of the CDF highlights differences in other measures of shape.

**Fig 2:**
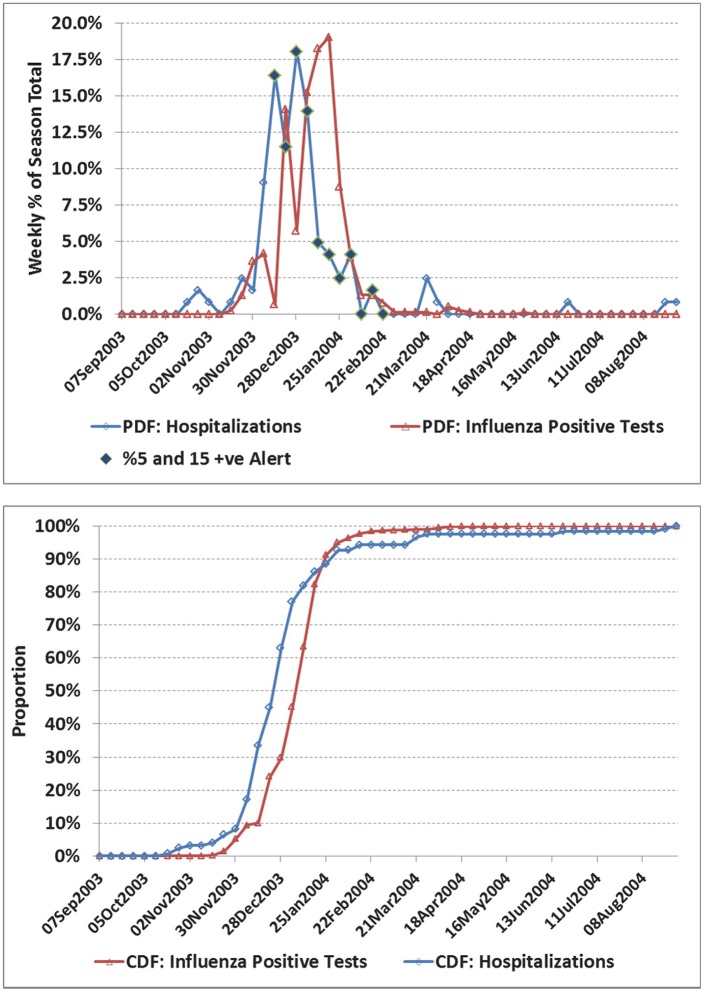
a) Weekly number of influenza positive tests and influenza admissions to hospital for the Atlantic region, 2003/04 season. b) Corresponding cumulative distribution functions (CDF). The two time series are in fair agreement with a correlation coefficient (*r*) of 0.55 (95% CI: 0.33, 0.72). Hospital admissions led by an average of 6 days. Still, the proposed alert covering 11 weeks included 77% of the admissions. An earlier alert would have been more helpful as admissions for the first week of the alert period were already close to the maximum, and the pre-peak alert period is 2 weeks.

**Fig 3:**
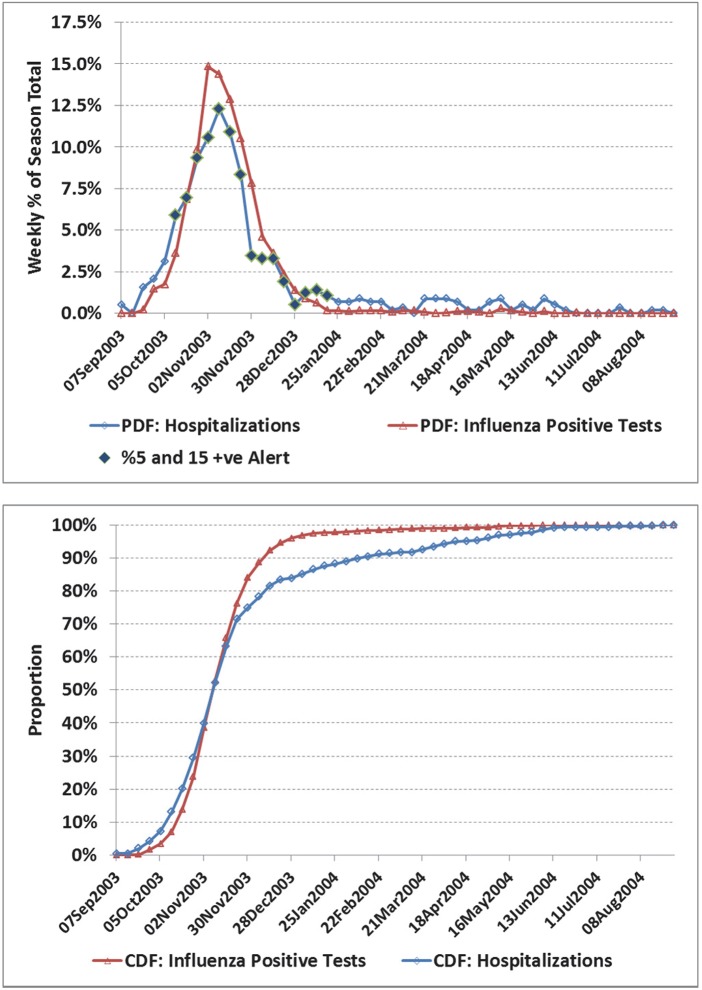
a) Weekly number of influenza positive tests and influenza admissions to hospital for the Prairies region, 2003/04 season. b) Corresponding cumulative distribution functions (CDF). The two time series are in close agreement with a correlation coefficient (*r*) of 0.97 (95% CI: 0.94, 0.98). However, influenza hospital admissions continued for many months after the epidemic subsided in this region, and the impact of these later admissions is highlighted by the CDF comparison. As a result, the average date of hospital admissions lagged influenza positive tests by an average of 12 days. This season is of interest due to the early epidemic peak (week of Nov 9, 2003). The pre-peak alert period is 4 weeks (the first alert was set based on laboratory data for the week of Sept 28, with the alert period starting operationally in the week of Oct 12), well ahead of peak influenza activity for the region, thereby providing significant advanced warning at a key time.

**Fig 4:**
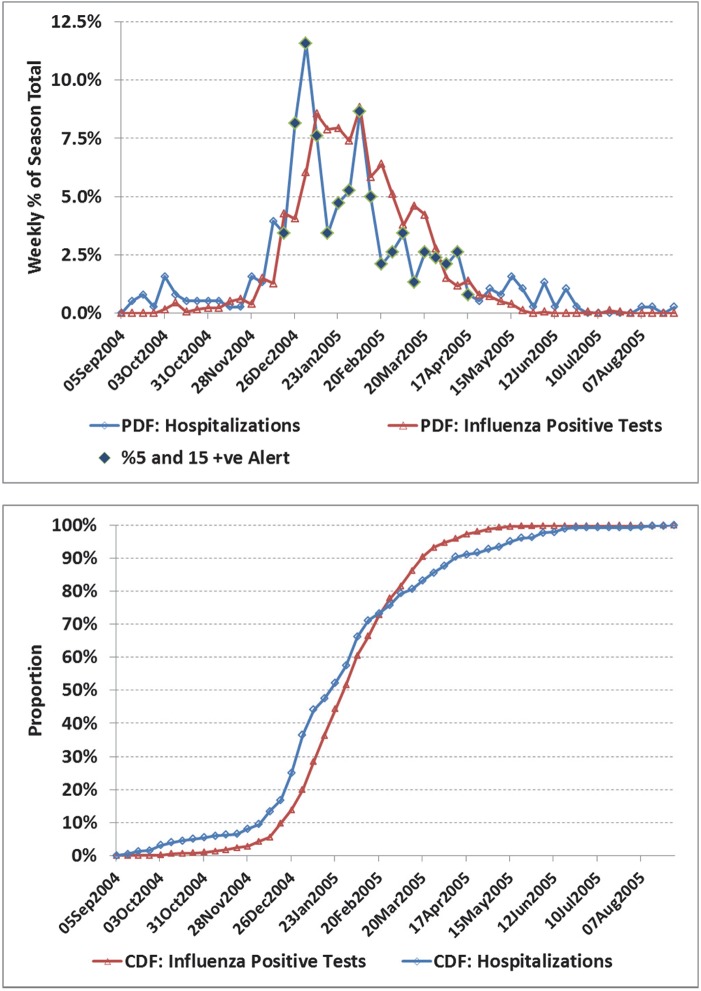
a) Weekly number of influenza positive tests and influenza admissions to hospital for the Prairies region, 2004/05 season. b) Corresponding cumulative distribution functions (CDF). The two time series are in fair agreement with a correlation coefficient (*r*) of 0.80 (95% CI: 0.67, 0.88). Hospital admissions led influenza positive tests by an average of 4 days (95% CI: -2.0, 9.9) though this difference was not statistical significant. The proposed alert covered 18 weeks and included 78% of the admissions. Again the viral identification data is more concentrated during periods of peak influenza activity than influenza hospitalizations.

**Fig 5:**
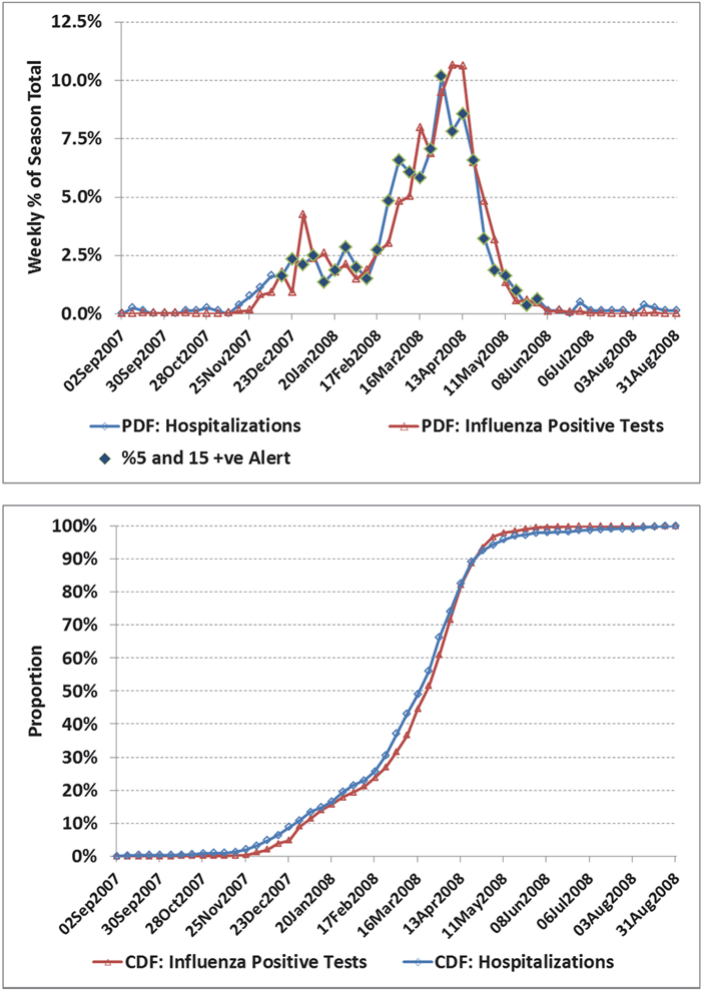
a) Weekly number of influenza positive tests and influenza admissions to hospital for the Ontario, 2007/08 season. b) Corresponding cumulative distribution functions (CDF). Considering that 42% of viral isolates were influenza B, the two time series are in close agreement with a correlation coefficient (*r*) of 0.95 (95% CI: 0.92, 0.97). As a result of multiple circulating strains, the alert was triggered 15 weeks before peak influenza admissions and remained on for 25 weeks. Influenza A (A/Solomon Islands/03/2006(H1N1)) was responsible for the initial increase in hospitalizations in January with A/Brisbane/10/2007 (H3N2) and B strain (B/Florida/4/2006 (belonging to the B/Yamagata lineage)) circulating in late spring.

**Fig 6:**
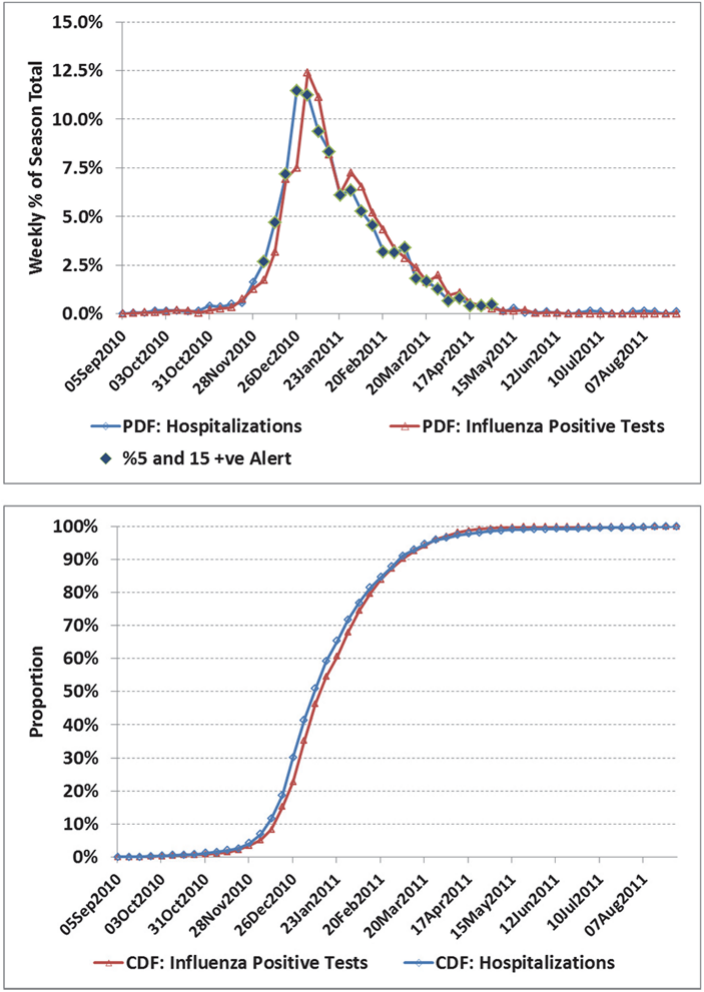
a) Weekly number of influenza positive tests and influenza admissions to hospital for the Ontario, 2010/11 season. b) Corresponding cumulative distribution functions (CDF). In this example, influenza admissions peaked in the last week of 2010 and first week of 2011, a time when resource planning can be more critical. The alert set based on laboratory data for the week of Nov 21 and available operationally the week of Dec 5, would have provided a 3 week notice of peak influenza activity.

The alert was set for week *t* if at least 15 influenza positive tests were observed for week *t* and the corresponding positivity rate was at least 5% (i.e. at least 300 specimens were tested in week *t* and 15 or 5% or more were positive). At any point in time, the most current influenza surveillance report is usually available for the period dated 2 weeks earlier. To allow for this delay in reporting and processing, a 2 week operational delay was assumed. That is, if the first alert was set based on laboratory reports for week 1, we assumed that the alert would be announced early in week 3, and preparations could begin in week 3. As gaps were more likely to occur at the beginning or end of the alert period when numbers were small, we waited one week before turning the alert off in order to improve the continuity of the alert period. The influenza hospitalizations for week *t*+2 (date of admission) were considered to be included in the alert period if the alert was set based on the virological data for week *t* or *t*-1. The length of the alert period is the time from the first to last alerted week (including any gaps). The length of the pre-peak period was calculated from the presumed week of first announcement (week of the first alert +2) to the week with the seasonal maximum number of hospitalizations by week of admission. All statistics were calculated based on the alert status as would have been reported in the most recent surveillance report available at the time of hospitalization. As well, the alerted weeks flagged in Figs 1–6 were adjusted for this 2 week operational delay. The first and last alerted weeks do not necessarily correspond to the beginning and end of the epidemic period as generally defined elsewhere to be periods in excess of what is normally expected (usually in reference to ILI surveillance). In using virological data for the alert, the ultimate objective is to provide some advanced warning of an emerging epidemic prior to observing an excess case load.

### Ethics Statement

This study was conducted in accordance with the principles expressed in the Declaration of Helsinki. Data provided to the Public Health Agency of Canada were collected under the Public Health Agency of Canada Act and were used in agreement with policy and regulations related to the publication of information related to public health. Identifying information was not available to this study. Hence, ethics approval was not required.

## Results

Over the study period, there were 11,070 influenza hospitalizations and 52,715 influenza positive test reports of which 12,550 (24%) were for influenza B. The ratio of positive tests to admissions was 4.8:1. As an influenza diagnosis could have been based on a point of care test, or more than one laboratory test could be associated with one admission, the exact relationship between the number of weekly influenza positive tests reported to *FluWatch* by laboratories and the number of patients admitted to hospital with a confirmed influenza diagnosis is not known.

### Lead-lag and other timing differences

Overall, influenza hospitalizations led laboratory positive tests an average of only 1.6 (95% CI: -1.5, 4.7) days. Though the average difference was not statistically significant, the difference in timing was statistically significant at the significance level of 0.01 in 8 out of 28 epidemic season and this difference exceeded 1 week in 5 seasons. After accounting for multiple comparisons, this level of detection remains highly significant (p-value<0.0001). The estimated variance was higher for hospitalizations than for influenza positive tests in all 28 periods analyzed, and statistically significant in most. The Two Sample Kolmogorov-Smirnov Test was significant at the significance level of 0.01 in 10 out of 28 of the periods analyzed (Table 1). From the perspective of public health, there was very close agreement in the weekly distribution of influenza positive tests and the number of patients admitted to hospital with influenza, though, the distribution of hospital admissions was spread over a slightly longer period, i.e. the tails of the distribution were noticeably longer. The comparison of two weekly indicators of influenza activity over one season often identified distinct and statistically significant differences, similar to differences previously observed between geographically adjacent population centers [21].

**Table 1:** Average Differences in Timing between the Week of Hospital Admission with Influenza and Report of Laboratory Confirmation of Influenza in Specimens Sent for Testing.

Region	Season	Average Date of Hospital Admission	Average Date of Influenza Positive Test Report	Difference in Days^1^	95%CI	F: Ratio of Variances	P-value (F): Equal Variances	t-value of Difference: Unequal Variance	p-value (t-test)	p-value KS
Atlantic	2003/2004	1-Jan-04	8-Jan-04	-6	(-13.9, 1.8)	4.31	< .0001	-1.54	0.1269	< .0001
Atlantic	2004/2005	9-Feb-05	9-Feb-05	-1	(-6.8, 5.6)	3.47	< .0001	-0.18	0.8557	0.0147
Atlantic	2005/2006	7-Mar-06	4-Apr-06	-28	(-46.6, -8.5)	8.25	< .0001	-2.90	0.0054	0.0453
Atlantic	2006/2007	4-Mar-07	8-Mar-07	-4	(-11.1, 3.7)	2.40	< .0001	-0.99	0.3221	0.0117
Atlantic	2007/2008	21-Mar-08	19-Mar-08	2	(-6.2, 10.3)	1.57	0.004	0.50	0.6188	0.3803
Atlantic	2008/2009	15-Feb-09	1-Mar-09	-15	(-24.2, -5.1)	2.64	< .0001	-3.07	0.0031	0.0384
Atlantic	2010/2011	28-Feb-11	1-Mar-11	-1	(-5.7, 3.3)	1.80	< .0001	-0.52	0.6064	0.5304
Ontario	2003/2004	23-Dec-03	24-Dec-03	-1	(-2.6, 1.3)	2.35	< .0001	-0.65	0.5154	< .0001
Ontario	2004/2005	17-Feb-05	23-Feb-05	-7	(-8.9, -4.6)	1.39	< .0001	-6.17	< .0001	< .0001
Ontario	2005/2006	14-Mar-06	18-Mar-06	-5	(-8.8, -0.6)	2.34	< .0001	-2.26	0.0241	0.1179
Ontario	2006/2007	6-Feb-07	13-Feb-07	-7	(-10.9, -3.8)	1.91	< .0001	-4.10	< .0001	< .0001
Ontario	2007/2008	11-Mar-08	14-Mar-08	-3	(-6.6, 0.5)	1.36	< .0001	-1.70	0.0900	0.0066
Ontario	2008/2009	15-Feb-09	21-Feb-09	-6	(-9.2, -1.9)	1.65	< .0001	-2.95	0.0033	0.0428
Ontario	2010/2011	19-Jan-11	21-Jan-11	-3	(-4.5, -0.8)	1.27	< .0001	-2.80	0.0052	< .0001
Prairies	2003/2004	27-Nov-03	15-Nov-03	12	(7.5, 17.3)	3.86	< .0001	4.98	< .0001	< .0001
Prairies	2004/2005	30-Jan-05	3-Feb-05	-4	(-9.9, 2.0)	2.31	< .0001	-1.31	0.1896	< .0001
Prairies	2005/2006	1-Mar-06	21-Feb-06	8	(1.8, 14.4)	2.41	< .0001	2.51	0.0125	0.0596
Prairies	2006/2007	14-Feb-07	31-Jan-07	14	(5.8, 22.6)	1.90	< .0001	3.33	0.0010	0.0034
Prairies	2007/2008	18-Feb-08	19-Feb-08	-1	(-6.4, 5.2)	2.29	< .0001	-0.20	0.8401	0.0197
Prairies	2008/2009	24-Feb-09	28-Feb-09	-4	(-9.6, 1.4)	1.39	0.001	-1.48	0.1406	0.5808
Prairies	2010/2011	19-Feb-11	14-Feb-11	5	(0.3, 9.0)	1.15	0.043	2.08	0.0377	0.3136
BC	2003/2004	16-Dec-03	12-Dec-03	4	(-3.1, 11.6)	1.78	< .0001	1.14	0.2544	0.2350
BC	2004/2005	31-Jan-05	30-Jan-05	1	(-13.2, 15.3)	2.29	< .0001	0.15	0.8840	0.0326
BC	2005/2006	15-Feb-06	11-Feb-06	5	(-5.7, 15.2)	1.96	< .0001	0.91	0.3668	0.0004
BC	2006/2007	17-Feb-07	15-Feb-07	2	(-8.5, 11.5)	2.37	< .0001	0.30	0.7658	0.5543
BC	2007/2008	14-Feb-08	20-Feb-08	-5	(-14.7, 4.0)	2.19	< .0001	-1.14	0.2576	0.0112
BC	2008/2009	18-Feb-09	21-Feb-09	-3	(-11.1, 5.1)	2.15	< .0001	-0.73	0.4643	0.6505
BC	2010/2011	20-Feb-11	15-Feb-11	5	(-2.7, 11.8)	1.75	< .0001	1.23	0.2196	0.2732
	Average			-1.6	(-4.7, 1.5)					

^1^ A positive difference indicates that the virological results led hospitalizations.

### External validation of the Alert

On average, 80% of hospitalizations were covered by the regional alert period adjusted for a 2 week operational delay. The average length of the alert period was 16 weeks and a gap in the alert period occurred twice. For the 2003/04 A/Fujian/411⁄02 (H3N2) season when a single strain accounted for 95% of the influenza viral identifications, the average alert period was 12.8 weeks. More recently, at least two strains circulated in significant numbers each season, which accounts for the longer alert period. Coverage rates were lowest in jurisdictions and seasons with a smaller number of influenza positive tests (Table 2). For 20 out of the 28 (71%) seasons, the first alert was signalled at least 3 weeks in real time prior to the week with the maximum number of influenza hospital admissions.

**Table 2:** Alert Characteristics.

Region	Influenza Season	% of Influenza Hospitalizations Covered by the Alert Period	# of Weeks Alerted	% Influenza B Isolates	Annual # of Influenza Positive Tests	Annual # of Influenza Confirmed Hospitalizations	# Weeks from 1st Alert to Peak Hospitalizations^1^
Atlantic	2003/04	77.0%	11	1.8%	767	122	2
Atlantic	2004/05	60.4%	10	4.4%	885	182	0
Atlantic	2005/06	68.5%	14	66.1%	454	54	4
Atlantic	2006/07	64.6%	12	3.9%	623	96	1
Atlantic	2007/08	89.6%	16	58.8%	775	77	7
Atlantic	2008/09	69.2%	11	29.3%	598	65	2
Atlantic	2010/11	85.2%	17	8.6%	1,652	291	3
Ontario	2003/04	87.2%	13	1.1%	3,729	1226	3
Ontario	2004/05	90.6%	19	28.0%	3,874	1301	6
Ontario	2005/06	83.5%	17	48.3%	1,962	424	2
Ontario	2006/07	86.4%	19	1.9%	2,432	632	6
Ontario	2007/08	93.1%	25	42.0%	4,317	806	15
Ontario	2008/09	89.3%	16	53.9%	2,431	413	7
Ontario	2010/11	94.3%	22	11.0%	7,750	1982	3
Prairies	2003/04	80.3%	15	0.2%	2,409	578	4
Prairies	2004/05	77.7%	18	14.5%	1,802	381	2
Prairies	2005/06	82.3%	20	44.0%	2,093	334	6
Prairies	2006/07	69.4%	17	3.5%	1,598	219	8
Prairies	2007/08	79.4%	18	48.2%	2,708	345	6
Prairies	2008/09	86.4%	13	32.7%	1,631	206	7
Prairies	2010/11	93.5%	24	32.8%	2,861	490	13
BC	2003/04	74.0%	12	0.2%	814	150	2
BC	2004/05	55.7%	13	11.4%	579	97	1
BC	2005/06	77.2%	19	42.9%	772	92	10
BC	2006/07	70.2%	12	6.1%	653	94	4
BC	2007/08	82.3%	21	44.7%	1,066	113	7
BC	2008/09	84.2%	14	23.5%	820	120	3
BC	2010/11	81.1%	15	27.4%	660	180	8
Average		79.7%	16.2	24.7%	1883	395	5
Average of 2003/04 Season^2^		79.6%	12.8	0.8%	1930	519	3

Notes

^1^ After accounting for a 2 week operational delay. In 12 out the 28 regional seasons, the alert should have been available at least 3 weeks before the peak in influenza hospitalizations.

^2^ A novel strain (A⁄Fujian⁄411⁄02) emerged in the 2003/04 season and dominated the season. Prompt alerts that are provided before the peak are more crucial in seasons when a single strain dominates. The alert period will be shorter when a single strain circulates.

For selected seasons, the weekly distribution of influenza-confirmed hospitalizations and influenza positive laboratory tests are shown in Figs 1 through 6 with the alerted weeks marked with a solid diamond. Note that the alerted weeks were adjusted for a 2 week operational delay to reflect the most recent alert status available during the corresponding week of admission to hospital. The time series used to set the weekly alerts (the weekly number and percent of laboratory tests positive for influenza) are not shown. The accompanying CDF plots illustrate the cumulative differences in timing over the full season that form the basis of the Two-Sample Kolmogorov-Smirnov Test. Examples were selected primarily from the two regions with the highest number of influenza positive tests, as with a larger sample size visual differences were more likely to correspond to statistically significant differences.

In the 2003/04 season a novel strain (A⁄Fujian⁄411⁄02) emerged to dominate the season (Fig 1). Agreement was good between the two curves. The alert was first set based on virological data for the week of Nov 16, and available for planning 2 weeks later (week of Nov 30), or 3 weeks before the peak in influenza hospitalizations (week of Dec 21). In Fig 2, the correlation between curves was poorer (*r* = 0.55), though coverage was still good (77%). However, the alert status was on for only 2 weeks in real time before the peak in influenza hospitalizations. In the 2003/04 season, the A⁄Fujian⁄411⁄02 strain emerged very early in the season in the Prairies (Fig 3). An unusually long tail for hospitalizations resulted in an estimated average lag of 12 days (95% CI: 7.5, 17.3), though the epidemic midpoint (CDF = 50%) occurred in the week of Nov 9 for both time-series. A pre-peak alert period of 4 weeks (set based on laboratory data for the week of Sept 28, and reportable in the week of Oct 12), would have provided significant advanced warning. Fig 4 illustrates the variation corresponding to a mixed season (A/Fujian/411/2002(H3N2) and A/California/7/2004) with two separate peaks. The alert period was long (18 weeks), though the pre-peak warning was short (2 weeks). Fig 5 is an example of a season where two influenza A strains (A/Solomon Islands/03/2006(H1N1) followed by A/Brisbane/10/2007 (H3N2)) and a B strain (B/Florida/4/2006, B/Yamagata lineage) circulated. The epidemic curves are very similar (*r* = 0.95), however the pre-peak alert period was 15 weeks. Fig 6 is an example where peak influenza activity occurred over the last week of December and first week of January, a time when advanced warning would be helpful for resource planning in the hospital setting. In this example, the alert provided a 3 week pre-peak warning period.

## Interpretation

This study shows that there was close agreement in the timing of the two indicators of influenza activity at the regional level: the number of laboratory tests reported positive for influenza and the number of admissions to hospital with a confirmed influenza diagnosis based on date of test and date of admission. Though there was no evidence that one indicator consistently led the other, subtle differences in timing were identified. Laboratory positive tests were slightly but significantly more concentrated during periods of peak activity, while a slightly higher proportion of hospitalizations occurred outside the peak period, as seen by the longer tails in the distribution figures. It is possible that viral testing is less frequent when influenza activity is low.

Also surprising was the large number of epidemic curve comparisons for which either the laboratory or hospitalization data led the other time series by more than 1 week. In some cases this difference could be attributed to the longer tail in the hospitalization data when the epidemic peaked early in the season (Fig 3). In this example the epidemic midpoint offered a more robust measure of lead/lag differences during the period of peak activity. Differences in testing frequencies and procedures between hospitals, clinical practices and health regions, as well as differences of a couple of weeks in the timing of peak activity within the region [21] could explain the irregular differences in timing.

An alert based on 5% positivity of laboratory tests and 15 positive tests provided good coverage of influenza-confirmed hospital admissions and a reasonable warning period in the 2003/04 season when the epidemic emerged very early and a single strain dominated. Increasing the threshold to 15% positivity reduced the average length of the alert period only slightly by 1 to 2 weeks and coverage from 80% to 75%. However, a reduction in the pre-peak warning period of 1 to 2 weeks could have a more significant impact on operations.

The threshold of 15 positive tests in one week is expected to be reached 5 weeks (3 weeks in real time) before the peak/epidemic midpoint in 90% of seasons if at least 800 positive tests are reported annually for the strain responsible for the peak. This estimate is based on previous estimates of the shape of the empirical epidemic curve [14] (the week five weeks before the epidemic midpoint accounted for approximately 2.5% of annual tests during seasons with a single dominant strain). As the number of positive tests nearly doubled from one week to the next during the exponential growth phase, reducing or increasing the threshold by a factor of two should advance or delay the start of the alert period by 1 week. However, small numbers of positive tests may represent clusters (from an institutional outbreak), and evidence of over-dispersion in influenza data is common. Though regions with fewer influenza confirmations could use a lower threshold, this would increase the risk of setting the alert very early based on strains circulating in the pre-epidemic period.

### Comparison with other studies

Despite numerous approaches to identify periods of influenza activity, thresholds are still usually set based on visual inspection. Even approaches based on complex statistical techniques usually require some pre-determined threshold to be nominated, again often by inspection [1]. A recent Delphi study used expert opinion (ie, visual inspection) to provide ground truth for algorithmic research [22]. In this study the focus was on identifying an alert that would provide some warning in real time prior to the epidemic peak for resource planning purposes and one that would include a large proportion of all confirmed influenza hospitalizations to be used, for example, to determine the period of empirical anti-viral treatment, as appropriate [23,24]. Noting that the onset of the influenza activity usually goes undetected for many weeks before the number of influenza cases is sufficiently large to be detected through methods used to identify excess morbidity or mortality or via laboratory testing, Cowling and colleagues [11] identified a similar objective of quickly generating an alarm before the start of the peak season.

Following the success of Google Flu Trends in identifying a leading indicator for weekly ILI consultation rates, a number of studies have shown strong correlations between various time-series based on other administrative data or social media and conclude that results are promising [5]. However, others have noted that the degree of correlation was highly variable among regions [2,6]. Our results agree more strongly with the latter conclusion.

Olson and colleagues used clinical ILI surveillance data as ground truth to assess the GFT estimates. However, despite observing strong correlations, they also identified substantial differences between the two weekly time series and concluded that search query data was no substitute for timely clinical or laboratory surveillance data [10]. As confirmed in our recent study of emergency department visits [25], it seems inappropriate to treat ILI surveillance as the ground truth for influenza activity when virological time-series are available. Ortiz and colleagues [26] also noted that GFT was more closely correlated with ILI consultation rates than laboratory-confirmed influenza and hence concluded that of the three time series, virologic surveillance is the most critical to the understanding of influenza activity.

Studies that have used the lag with maximum cross-correlation have found considerable variation in the estimates of timeliness of peak influenza activity for different data sources [3]. Our study confirms that some differences in timing, which could be due to a lack of geography representativeness [21], likely exist, though any noted differences are not likely to be reproducible. Since the difference between the maximum and the second largest cross-correlation coefficient was often not statistically significant, our recommendations for assessing differences in lead/lag timing of periods of peak influenza activity is to use differences in the average date of infection or, preferably, the epidemic midpoint, as the average is more sensitive to differences in the off-season (or tails of the distribution).

Ginsberg and colleagues noted that Google web search queries could produce ILI estimates that were consistently 1–2 weeks ahead of CDC ILI surveillance reports because search query data could be processed quickly [7]. As the time from symptom onset to hospital admission averaged 4 days during the 2009 pandemic [27], and positive findings are less likely in persons presenting for medical care more than a week after symptom onset [28], it appears unlikely that secondary data sources could be found that would consistently lead the influenza epidemic by more than the reporting and operational delay of 1–2 weeks. There are a few possible exceptions that we have noted elsewhere: though lead/lag times between adjacent health regions have not been consistent, the Atlantic region in Canada has shown a tendency to lag other regions in Canada and the United States [21]; and influenza infections in persons aged 15–19 and 20–24 years have been shown to lead other age groups by up to 1 week [29]. As this age group is more web savvy, web-based participatory surveillance projects [30] may be able to tap into this lead group. Because of the high baseline level for self-reports of influenza symptoms [31], it is unlikely that the age-specific alert based on self-reports of ILI would be triggered earlier, however, youth reports may be used to signal the timing of peak activity, or more specifically the end of exponential growth phase in the general population.

Reich and colleagues [32] is the only other study we are aware of to use similar operational performance criteria. They illustrated that alerts based on influenza-confirmed hospitalizations could be set at the hospital level, with thresholds in the range of 3 to 5 hospitalizations or approximately 2.5% of the annual total. Immediate access to hospital data would increase the timeliness by 1 week, though the small numbers would increase random variation. A threshold of 5 positive tests is too small for virological data from the general population as tests associated with an institutional outbreak in the pre-epidemic period or other sources of clustering could trigger an early alert. The regional and local (hospital) approaches are complimentary, and in both cases, preparations for a surge in influenza cases would have to start based on a relatively small case load.

### Limitations

We used historical data on confirmed influenza hospitalizations to assess the performance of an alert base on 5% positivity and at least 15 positive tests in one week at the regional level as an indicator of influenza activity. This assessment has a number of limitations. Confirmation of an influenza infection either through laboratory or point of care testing is still limited, so that the alert was set at a regional level–a geographic scale that is at times too large to ensure synchronicity within each region [21]. Since the relationship between laboratory tests and the burden of disease (influenza-attributed hospitalizations, for example) may vary from season to season [25], the number of laboratory confirmations is not a direct measure of the disease burden. However, as the number of confirmed influenza hospitalizations is only a fraction of all hospitalizations attributable to influenza [16,33] and only a small proportion of emergency department visits attributed to an influenza infection were given an ILI diagnosis [25], direct measures of the disease burden are not available. Though an indirect measure, regression models have been successful in estimating the annual burden attributable to influenza [16]. Though we did not compare the alert period generated by the virological data head-to-head with weekly ILI surveillance data due to data limitations, viral identifications would still be considered the gold standard to assess whether any excess in ILI consultations is likely due to influenza. We did not provide confidence intervals for many of the summary measures, such as the expected coverage rate associated with this threshold rule, as the characteristics of each season are highly variable and it is unclear to what extent the results can be generalized to future seasons.

## Conclusions

In summary, virological data for four regions in Canada provided a reasonable pre-peak warning and indicated the period of influenza activity that covered most influenza hospitalizations. There is no consensus on a gold standard to define the period of influenza activity, and it seems unlikely that there will ever be one. More likely, the influenza indicator will be used in conjunction with the specific resource data in question, and the pre-peak alert will be used by health care resource managers as a reminder to monitor resources more closely especially over the next couple of weeks. Alerts based on more complex time-series, such as ILI consultations, may provide reasonable performance; however, cross-validation is required to assess any performance advantages over virological data. This evaluation approach should be adaptable to alerts based on other surveillance time series, or the criteria could be modified to suit specific resource management issues. It is, however, important to conduct the performance evaluate over many epidemics, especially when evaluating lead time differences, as each epidemic is a unique mix of multiple strains of varying timing and severity.

## Supporting Information

S1 Table Influenza Tests, FluWatch, Public Health Agency of Canada. (XLSX)
